# Bioinformatics Analysis Identifies TNFRSF1A as a Biomarker of Liver Injury in Sepsis TNFRSF1A is a Biomarker for Septic Liver Injury

**DOI:** 10.1155/2022/1493744

**Published:** 2022-10-15

**Authors:** Shangxun Zhou, Wei Zhao, Junjie Li, Yang Huang, Jing Yang, Qianmei Wang, Yunyun Xu, Chujun Duan, Yutong Wang, Wen Yin

**Affiliations:** Department of Emergency, Xijing Hospital, Air Force Medical University, Xi'an 710032, Shaanxi, China

## Abstract

Sepsis is a severe disease with high mortality, and liver injury is an independent risk factor for sepsis morbidity and mortality. We analyzed co-differentially expressed genes (co-DEGs) to explore potential biomarkers and therapeutic targets for sepsis-related liver injury. Three gene expression datasets (GSE60088, GSE23767, and GSE71530) were downloaded from the Gene Expression Omnibus (GEO). DEGs were screened between sepsis and control samples using GEO2R. The association of these DEGs with infection and liver disease was analyzed by using the CTD database. GO functional analysis, KEGG pathway enrichment analysis, and protein-protein interaction (PPI) network analysis were performed to elucidate the potential molecular mechanism of DEGs. DEGs of different tissues in GSE60088 were analyzed again to obtain specific markers of septic liver injury. Mouse model of sepsis was also established by cecal ligation and puncture (CLP), and the expression of specific markers in liver, lung, and kidney tissues was analyzed using Western blot. Here, we identified 21 DEGs in three datasets with 8 hub genes, all of which showed higher inference scores in liver diseases than bacterial infections. Among them, only TNFRSF1A had a liver-specific differential expression. TNFRSF1A was also confirmed to be specifically reduced in septic liver tissues in mice. Therefore, TNFRSF1A may serve as a potential biomarker for septic liver injury.

## 1. Introduction

Sepsis is an infection-induced systemic inflammatory response syndrome. It is a common complication of patients with severe trauma, shock, and critical diseases and a leading cause of death for patients in the intensive care unit (ICU). The occurrence of sepsis is associated with the hypersensitivity cascade and excessive inflammatory mediators [1–3]. Identification of sepsis-related biomarkers is critical for the diagnosis to distinguish the severity and develop a treating strategy of sepsis. Increasing evidence has demonstrated that multiple genes are involved in sepsis progression and organ damage [4–7]. Zheng et al. identified fourteen long noncoding RNAs that could be used to diagnose sepsis patients with insignificant clinical manifestations [8]. Studies uncovered that the ribosome-related genes TLCD4, PRSS30P, and ZNF493 had a moderate performance to identify sepsis-induced acute respiratory distress syndrome (ARDS) in sepsis patients [9, 10]. Five genes (NKG7, SPTA1, FGL2, RGS2, and IFI27) have been proved to be potential biomarkers for sepsis-induced ARDS and exert crucial roles in the occurrence and development of sepsis [11]. Sepsis may also induce acute kidney injury (AKI), and studies showed that VMP1, SLPI, PTX3, TIMP1, OLFM4, LCN2, and S100A9 genes were markedly correlated with the development and progression of septic-shock-associated AKI [12]. Sepsis-caused failure in different tissues has different mechanisms. Serving as an important host defense organ through bacterial clearance, acute phase proteins, cytokine generation, and metabolic adaptation to inflammation, the liver is one of the most vulnerable organs in patients with sepsis. Sepsis-induced liver injury or dysfunction is considered as a strong independent predictor of mortality in ICU (up to 54-68%) [13–15]. However, the genes involved in sepsis-induced liver injury remain unclear.

Microarray technology and bioinformatics analysis have been widely used to screen for genetic alterations at the genome level, contributing to the identification of differentially expressed genes (DEGs) and functional pathways in disease models. Analysis of microarray data in a septic liver injury model can build gene networks and screen for potential key molecular targets to provide a new understanding of the pathogenesis of septic liver injury and a potential strategy for clinical treatment [16, 17]. In this study, we downloaded and analyzed three mRNA microarray datasets from the Gene Expression Omnibus (GEO) database to obtain DEGs between normal and septic liver tissues. The GO functional analysis, KEGG pathway enrichment analysis, and protein-protein interaction (PPI) network analysis were performed, and a total of 21 DEGs and 8 hub genes were identified, of which only TNFRSF1A was critical for the specificity of septic liver injury, which could be a candidate biomarker.

## 2. Methods

### 2.1. Microarray Data

GEO (https://www.ncbi.nlm.nih.gov/geo) is a public functional genomics repository containing global gene expression data and microarrays [18]. Three gene expression datasets, GSE60088 [19], GSE23767 [20], and GSE71530 [21], were downloaded from public GEO (Affymetrix GPL570 platform, Affymetrix Human Genome U133 Plus 2.0). Among them, GSE60088 contains 5 sepsis samples and 3 control samples; GSE23767 includes 4 sepsis and 3 control samples; and GSE71530 contains 3 sepsis and 3 control samples. The datasets from septic and normal liver tissues were collected and screened for subsequent analysis.

### 2.2. Identification of DEGs

GEO2R (https://www.ncbi.nlm.nih.gov/geo/geo2r) was used to screen significant DEGs between sepsis and control liver samples. The application of *P*-values and Benjamini and Hochberg false discovery rates provided a balance between the discovery of statistically significant genes and false positive limits. The probe sets without any gene symbols or genes with multiple probe sets were excluded or averaged, respectively. A fold change (logFC) >1 and *P*-value <0.05 were considered statistically significant.

### 2.3. PPI Network Construction and Analysis

PPI networks were predicted using an online database searching tool (STRING; https://www.string-db.org) to retrieve gene interactions [22]. Analysis of protein functional interactions provides insights into the inner mechanism of the related disease pathogenesis and development. In this study, the PPI network of DEGs was constructed by using the STRING, and interactions with a combined score >0.4 were considered to have statistical significance. Cytoscape software was applied to construct and visualize the molecular interaction network.

### 2.4. KEGG and GO Enrichment Analyses of DEGs

The Database for Annotation, Visualization, and Integrated Discovery 6.7 (DAVID; https://www.david.ncifcrf.gov) is an online bioinformatics database that integrates biological data and analysis tools to provide complete annotation information of functional genes and proteins for users [23]. GO functional analysis (including cellular composition [CC], biological process [BP], and molecular function [MF]) is a major bioinformatics tool that can classify gene expression and its related biological processes [24]. KEGG is a database resource that integrates large-scale molecular datasets using high-throughput techniques to understand their related functional pathways and biological systems [25]. To analyze the functions of screened DEGs, DAVID and the online bioinformatics database were used to perform biological analyses. *P* < 0.05 was considered statistically significant.

### 2.5. Associations of Common DEGs with Infection and Liver Disease

The Comparative Toxicogenomics Database (CTD; https://www.ctdbase.org/) is a public resource that describes the interactions between environmental chemicals and gene products and their relationship to disease [26]. We used these data to analyze and determine the association of common DEGs with infection and liver disease.

### 2.6. Mouse and Histological Analysis (Experimental Study)

Male C57BL/6 mice (6-8-week-old, weighing 20-25 g) from the Fourth Military Medical University (Xi'an, China) were housed under standard laboratory conditions. Mice were randomly divided into sham (*n* = 3) and CLP (*n* = 3) groups. The sepsis model was induced by CLP as previously described [27]. The experiment was repeated three times. Mice in the sham group were administrated with similar procedures without CLP. All mice were received 1 mL of normal saline in the abdominal cavity after surgery to compensate for fluid loss. While animals subjected to CLP appear healthy in the initial phase after the procedure, they begin to show clinical signs of sepsis at around 12 h following CLP, featuring malaise, fever, chills, piloerection, generalized weakness, and reduced gross motor activity. After 24 hours, the mice were sacrificed by decapitation, and the liver, lung, and kidney specimens were collected for hematoxylin and eosin (HE) staining. Briefly, the tissues were paraffin-embedded, sectioned at 5 *μ*m thickness, and stained with hematoxylin and eosin. The pathological sections were randomly observed by pathologists to analyze the pathological damage in each group. The animal study was performed following the Guide for the Care and Use of Laboratory Animals and approved by the ethics committee of the Xijing Hospital (approval number KY20193106).

### 2.7. Western Blot

Total proteins of the liver, lung, and kidney tissue samples from normal and septic mice were extracted using RIPA buffer (containing 1% PMSF and 1% protease inhibitors). BCA protein assay kit (Pierce, Rockford, USA) was used for protein quantification. Certain amounts of protein were loaded onto the SDS-PAGE gels and then transferred to PVDF membranes for Western blot analysis. After blocking, the membranes were incubated with the anti-TNFRSF1A primary antibody at 4°C overnight, followed by incubation with diluted HRP-conjugated secondary antibody (Pierce Biotechnology, Inc, Rockford, IL, USA) for 1 h at room temperature. Blots were visualized with ECL-Plus reagent (GE Healthcare, Piscataway, NJ). *β*-Actin antibody was used to confirm equal protein loading.

### 2.8. Statistical Analysis

Data analysis was performed using GraphPad Prism software (v.6.0; GraphPad software, La Jolla, CA, USA). Student's *t*-test was used. All animal experiments had at least three replicates. A value of *P* < 0.05 was considered statistically significant.

## 3. Results

### 3.1. Identification of DEGs in Septic Liver Tissue

**Figure 1:**
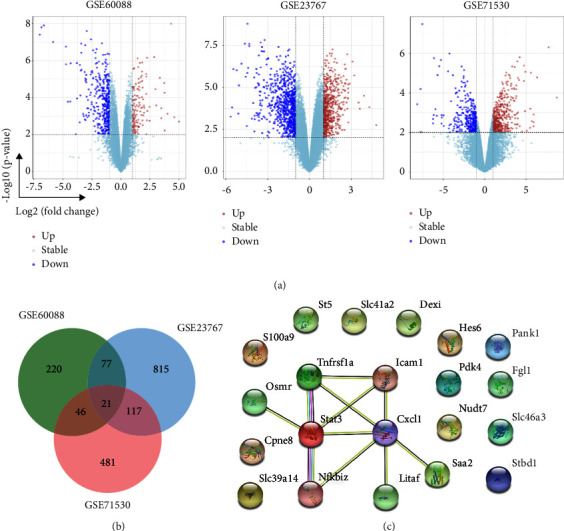
Identification of differentially expressed genes (DEGs) in septic liver tissues from the online database. (a) Distribution of DEGs in GSE60088, GSE23767, and GSE71530 datasets. (b) Venn diagram of the DEGs. There were 21 overlapping genes among the three datasets. (c) PPI network of 21 overlapping genes from STRING.

After normalization of the microarray results, there were 364, 1,030, and 665 DEGs identified in GSE60088, GSE23767, and GSE71530, respectively (Figure 1(a)). The overlap between the 3 datasets contained 21 genes, in which 15 genes were downregulated and 6 were upregulated in sepsis compared with the control (Figure 1(b)). A PPI network was constructed to show the interaction between these 21 genes (Figure 1(c)). Among them, eight of the 21 genes were closely interacted (Figure 1(c)), all of which were downregulated.  Table 1 shows the brief description of them.

### 3.2. Functional Enrichment and Disease Association Analysis of DEGs

**Figure 2:**
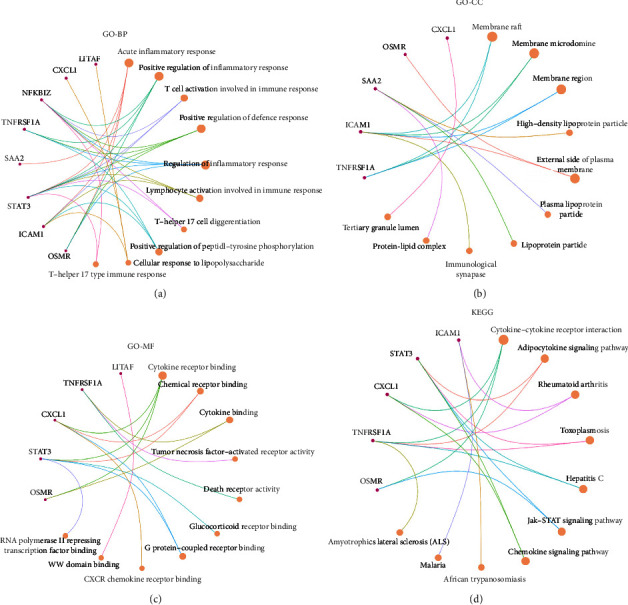
Functional enrichment pathway of the hub eight DEGs. (a-c) GO functional analysis of the genes, including pathways related to biological process (BP), cellular composition (CC), and molecular function (MF). (d) KEGG pathway analysis of the genes.

We further analyzed the functional and pathway enrichment of the eight DEGs through DAVID. Results from GO functional analysis indicated that the alteration of BP was mainly on the inflammatory and immune responses (Figure 2(a)). The changes of CC of DEGs focused largely on the membrane-related regions (Figure 2(b)), and MF alterations were mainly in the cytokine or chemical receptor binding (Figure 2(c)). KEGG pathway enrichment analysis showed that these DEGs were mainly involved in cytokine interaction, adipocytokine signaling, rheumatoid arthritis, toxoplasmosis, hepatitis C, JAK-STAT3 pathway, and chemokine signaling pathways (Figure 2(d)). The CTD database was applied to evaluate the association between the eight DEGs and infections and liver diseases. Results showed that all of them had greater inference scores associated with liver injury or disease than bacterial infections (Table 2).

### 3.3. Identification of TNFRSF1A as a liver-specific DEG in Sepsis

**Figure 3:**
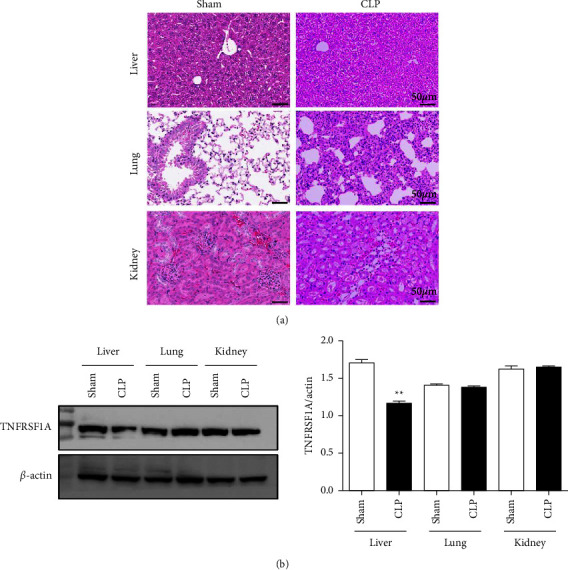
Identification of TNFRSF1A as a liver-specific DEG in sepsis. CLP-induced sepsis mouse model was established. (a) Representative HE staining images of the liver, lung, and kidney tissues of sham and CLP groups. Scale bar means 50 *μ*m. (b) Relative expression of TNFRSF1A in these animal septic tissues was detected through Western blot.  ^*∗*^ ^*∗*^*P* < 0.01 vs. Sham group.

**Figure 4:**
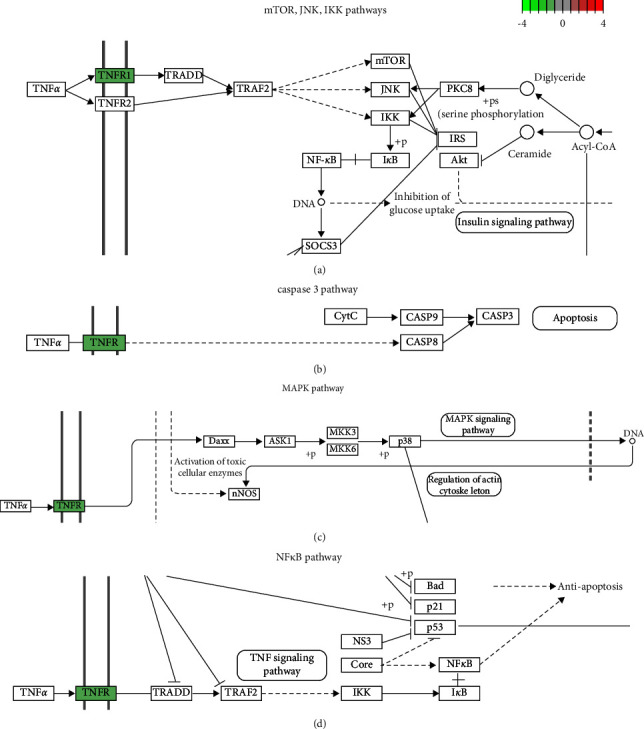
TNFRSF1A-related pathways in the KEGG database. It was involved in the mTOR (a), caspase 3 (b), MAPK (c), and NF*κ*B (d) pathways.

The eight DEGs were compared with the DEGs in the septic liver, kidney, and lung tissues of GSE60088, and it was found that only TNFRSF1A had a specific expression change in the liver (Table 3). Combined with the higher association of TNFRSF1A with liver-related diseases or injury than infections, it seemed that TNFRSF1A may be specific in liver disease. We further established CLP-induced sepsis mouse model. HE staining of the liver, lung, and kidney tissues of sham and CLP groups showed that all tissues were structurally disordered and had more or less inflammatory infiltration after CLP (Figure 3(a)). Liver tissues in sham were intact with normal and well-structured hepatic cells, while necrotic hepatocytes with extensive vacuolar degeneration and nuclear rupture were observed in the liver after CLP. The protein levels of TNFRSF1A in these animal septic tissues were detected through Western blot, which showed that TNFRSF1A was significantly downregulated in the liver tissues of septic mice compared with the sham. There was no big change between them in the lung and kidney (Figure 3(b)). Moreover, we searched TNFRSF1A in the KEGG database, which showed that TNFRSF1A, binding to TNF-*α*, mainly participates in the mTOR, MAPK, caspase 3, and NF*κ*B pathways that were related to the regulation of inflammatory responses and cell apoptosis (Figure 4).

## 4. Discussion

Despite extensive research in sepsis, there are still few biomarkers that can be used to effectively detect and treat sepsis [28]. Liver has a regenerative function and capability to withstand attack. In sepsis, the liver is a major site occurring inflammatory responses to defend bacterial endotoxins. Once liver dysfunction or failure happens, the damaged liver may cause severe systemic inflammatory responses spreading to other organs, leading to complication progression and even death [29–31]. Hence, the identification of liver damage-related genes could provide new targets and strategies for exploring the effects of the liver in sepsis and its related treatments.

Microarray assay is an effective method to screen novel biomarkers of disease and find genetic alterations in disease progression, which has been proved to be applicable in the study of septic biomarkers and organ damage [32]. In the current study, three databases GSE60088, GSE23767, and GSE71530 were found by screening the experimental data of sepsis complicated with liver injury in the GEO database. We applied microarray assay analysis and obtained 21 DEGs between septic liver tissues and normal ones, including 15 downregulated (ST5, NFKBIZ, PDK4, OSMR, STAT3, CPNE8, S100A9, TNFRSF1A, ICAM1, SLC39A14, SLC41A2, FGL1, CXCL1, LITAF, and SAA2) and 6 upregulated genes (HES6, STBD1, DEXI, PANK1, SLC46A3, and NUDT7). Among them, only 8 downregulated genes (OSMR, TNFRSF1A, ICAM1, STAT3, CXCL1, NFKBIZ, LITAF, and SAA2) interacted through the proteins they expressed. To analyze the association of these 8 key genes with sepsis and liver injury, we performed GO functional analysis and KEGG pathway enrichment analysis on them; GO functional analysis showed that they were mainly involved in the processes of acute inflammatory response, positive regulation of inflammatory response, activation of T cells in the immune response, and positive regulation of defense response. KEGG pathway enrichment analysis showed that they were enriched in the pathways associated with cytokine interactions. It was worth noting that TNFRSF1A, STAT3, ICAM1, and OSMR are involved in multiple biological processes and signaling pathways in each enrichment analysis. We speculated that the overlap in significantly enriched GO terms and KEGG pathway might represent vital pathways in the sepsis-induced liver injury. During sepsis, it is very likely that the liver plays a role in immune regulation and inflammatory clearance through one or more of these 4 key genes. Moreover, all these 8 genes showed higher correlation with liver diseases than bacterial infections in the CTD database.

However, these DEGs were not liver-specific. Through further analysis of dataset GSE60088 that contained DEGs of the septic liver, lung, and kidney, it was found that only TNFRSF1A was differentially liver-specific expressed among the 4 key genes. The effects of TNF-*α* can be exerted through two different receptors belonging to the TNF receptor superfamily. The type I receptor is TNFRSF1A, also known as p55, p60, CD120a, or TNFR1. TNFRSF1A is a 60 kDa transmembrane glycoprotein and expressed in almost all cells except for erythrocytes, but type II receptor mainly exists in immune cells, endothelial cells, and cells of the hematopoietic lineage. Both receptors can be activated by transmembrane TNF-*α*, but TNFRSF1A can also have functions by soluble TNF-*α* [33]. The activation of THFR induces the release of proinflammatory cytokines and chemokines. Other than that, TNFRSF1A possesses a cytoplasmic death domain (DD) that allows them to transduce regulated prodeath signals, leading to apoptosis or necrosis [34, 35]. Several studies have investigated and revealed the role of TNFR in the development of early and late renal failure, including diabetic nephropathy, renal angiosclerosis, acute renal transplant rejection, renal cell carcinoma, glomerulonephritis, sepsis, and obstructive renal injury [36]. In addition, TNFRSF1A mutation-caused tumor necrosis factor receptor-associated periodic syndrome (TRAPS) is the first and the only disease known to be caused by receptor structure mutation [37]. In sepsis, TNFRSF1A is a key participant during *Staphylococcus aureus* infections and is associated with the bacterial clearance from the spleen [38]. The significant increase of soluble TNFRSF1A in the circulation is closely related to sepsis. Using soluble TNFRSF1A to neutralize TNF reduces organ damage and mortality in sepsis rat [39].

In our experimental study, TNFRSF1A expression was detected in the liver, lung, and kidney tissues of sepsis mice, and only TNFRSF1A in the liver was significantly downregulated, which was consistent with the bioinformatics analysis. Hepatocytes from septic mice developed extensive vacuolar degeneration and nuclear rupture, which were closely related to the cytokine storm and inflammatory cascade triggered by sepsis. Membrane TNFRSF1A induces cellular inflammatory damage and apoptosis by participating in mTOR, JNK, IKK, caspase 3, MAPK, and NF-kB pathways. Therefore, the decreased TNFRSF1A protein expression in the liver tissue of the sepsis model combined with the downregulation of gene expression verified that TNFRSF1A may serve as a specific biomarker of septic liver damage and liver immunoregulation. Although the mortality of TNFRSF1A^−/−^ mice in sepsis was comparable with wildtype mice [40], it is still undeniable the important role of TNFRSF1A in septic liver injury. The extracellular domain of membrane-bound TNFR1 can be proteolytic-cleaved. Deng et al. confirmed that TNFR1 shedding in hepatocytes is through the iNOS-cGMP-TACE pathway to defend bacterial lipopolysaccharide [41], so intervention of this pathway may be beneficial for the early clinical cause of severe sepsis. Nevertheless, TNFRSF1A also participates in other pro- or anti-inflammatory pathways in liver cells. The KEGG database showed that TNFRSF1A involves in the mTOR and MAPK pathways to further regulate inflammatory responses, as well as caspase 3 and NF*κ*B pathways to control cell apoptosis, which maybe the inner mechanism of TNFRSF1A regulating liver immune defense and immunity adjustment. Our study indicated that not only the TNFRSF1A membrane protein in septic liver cells significantly decreased but also its gene expression had a specific decline. Its gene regulation mechanism remains to be investigated. Only by further exploring the duration, concentration, and related inflammatory factors of TNFRSF1A in the immunoregulation of sepsis, can we better understand its role in the occurrence and development of septic liver injury and provide potential insights and targets for the diagnosis and treatment.

In conclusion, our study showed that TNFRSF1A is closely associated with sepsis-induced liver injury, which provides a potential diagnostic signature for septic liver injury and a basis for exploring the roles the liver plays in defense homeostasis during sepsis. TNFRSF1A may serve as an intervention target to alleviate and treat sepsis in the future.

## Figures and Tables

**Table 1:** Description of the eight hub genes among the 21 overlapping genes in GSE60088, GSE23767, and GSE71530 datasets.

No	Gene symbol	Full name	Function
1	OSMR	Oncostatin M receptor	Binds IL31 to activate STAT3 and possibly STAT1 and STAT5
2	TNFRSF1A	Tumor necrosis factor receptor superfamily, member 1a	Receptor for TNFSF2/TNF-alpha and homotrimeric TNFSF1/lymphotoxin-alpha
3	ICAM1	Intercellular adhesion molecule 1	ICAM proteins are ligands for the leukocyte adhesion protein LFA-1
4	STAT3	Signal transducer and activator of transcription 3	Transcription factor that binds to the IL-6 responsive elements identified in the promoters of various acute-phase protein genes
5	CXCL1	Chemokine (C-X-C motif) ligand 1	Has chemotactic activity for neutrophils. Contributes to neutrophil activation during inflammation
6	NFKBIZ	Nuclear factor of kappa light polypeptide gene enhancer in B cells inhibitor, zeta	Inhibits NF-kappa-B activity without affecting its nuclear translocation upon stimulation. It is recruited to IL-6 promoters and activates IL-6 but decreases TNF-alpha production in response to LPS
7	LITAF	LPS-induced TN factor	May regulate through NFKB1 the expression of the CCL2/MCP-1 chemokine. May play a role in tumor necrosis factor alpha (TNF-alpha) gene expression
8	SAA2	Serum amyloid A 2	Major acute phase reactant. Apolipoprotein of the HDL complex

**Table 2:** Association of the eight hub genes with infection and liver diseases (from the CTD database).

Gene	Disease name	Disease ID	Inference score	Reference count
OSMR	Bacterial infections	MESH: D001424	11.47	4
	Bacteremia	MESH: D016470	6.67	5
	Chemical and drug-induced liver injury	MESH: D056486	181.83	996
	Liver diseases	MESH: D008107	97.81	80
	Liver failure, acute	MESH: D017114	44.32	190

TNFRSF1A	Bacterial infections	MESH: D001424	12.97	10
	Bacteremia	MESH: D016470	7.65	7
	Staphylococcal infections	MESH: D013203	15.66	112
	Chemical and drug-induced liver injury	MESH: D056486	373.55	1473
	Liver diseases	MESH: D008107	173.23	133
	Liver failure, acute	MESH: D017114	105.65	219

ICAM1	Bacterial infections	MESH: D001424	38.09	14
	Bacteremia	MESH: D016470	10.32	8
	Immune suppression	OMIM: 146850	22.44	4
	Staphylococcal infections	MESH: D013203	31.17	123
	Chemical and drug-induced liver injury	MESH: D056486	624.5	1763
	Liver diseases	MESH: D008107	253.83	168
	Liver failure, acute	MESH: D017114	145.9	251

STAT3	Bacterial infections	MESH: D001424	30.62	14
	Immune suppression	OMIM:146850	9.5	4
	Staphylococcal infections	MESH: D013203	15.89	113
	Chemical and drug-induced liver injury	MESH: D056486	468.99	1621
	Liver diseases	MESH: D008107	196.71	155
	Liver failure, acute	MESH: D017114	104.56	220

CXCL1	Bacterial infections	MESH: D001424	38.75	13
	Bacteremia	MESH: D016470	19.38	11
	Staphylococcal infections	MESH: D013203	27.98	126
	Chemical and drug-induced liver injury	MESH: D056486	492.25	1599
	Liver diseases	MESH: D008107	194.1	160
	Liver failure, acute	MESH: D017114	119.02	237

NFKBIZ	Bacterial infections	MESH: D001424	16.93	10
	Pneumococcal infections	MESH: D011008	11.06	4
	Staphylococcal infections	MESH: D013203	13.23	110
	Chemical and drug-induced liver injury	MESH: D056486	223.35	1151
	Liver diseases	MESH: D008107	118.82	94
	Liver failure, acute	MESH: D017114	71.4	189

LITAF	Bacterial infections	MESH: D001424	20.71	8
	Pneumococcal infections	MESH: D011008	11.09	4
	Staphylococcal infections	MESH: D013203	10.05	99
	Chemical and drug-induced liver injury	MESH: D056486	269.74	1285
	Liver diseases	MESH: D008107	145.5	101
	Liver failure, acute	MESH: D017114	80.37	205

SAA2	Bacterial infections	MESH: D001424	5.71	2
	Candidiasis	MESH: D002177	7.29	13
	Q fever	MESH: D011778	9.64	1
	Chemical and drug-induced liver injury	MESH: D056486	146.38	987
	Liver diseases	MESH: D008107	96.08	79
	Liver failure, acute	MESH: D017114	36.44	165

**Table 3:** TNFRSF1A has a specific expression change (√) in liver tissues after comparison with the DEGs in GSE60088.

Gene symbol	Liver	Lung	Kidney
OSMR	√	√	√
TNFRSF1A	√		
ICAM1	√	√	√
STAT3	√	√	√
CXCL1	√	√	√
NFKBIZ	√	√	√
LITAF	√	√	√
SAA2	√	√	

## Data Availability

The raw data supporting the conclusions of this article are available from the corresponding author upon request.

## Supplementary Materials

Supplementary Materials The abbreviations have been provided in Supplementary Table 1. The online version contains supplementary material available at the website of Genetics Research.
