# Supplementary material for: Bioinformatics Analysis Identifies TNFRSF1A as a Biomarker of Liver Injury in Sepsis TNFRSF1A is a Biomarker for Septic Liver Injury
Source: Genet Res (Camb). 2022 Oct 15;2022:1493744. doi: 10.1155/2022/1493744 (PMC9587912; doi:10.1155/2022/1493744)
Supplement: Supplementary Materials — The abbreviations have been provided in Supplementary Table 1. The online version contains supplementary material available at the website of Genetics Research. [file 1493744.f1.docx]

Supplementary Table 1. Abbreviations.

| co-DEGs | co-differentially expressed genes |
| --- | --- |
| GEO | Gene Expression Omnibus |
| CTD | Comparative Toxicogenomics Database |
| GO | Gene Ontology |
| KEGG | Kyoto Encyclopedia of Genes and Genomes |
| PPI | Protein-protein interaction |
| CLP | Cecal ligation and puncture |
| TNFRSF1A | Tumor necrosis factor receptor superfamily, member 1A |
| ICU | Intensive care unit |
| TLCD4 | TLC domain containing 4 |
| PRSS30P | Serine protease 30, pseudogene |
| ZNF493 | Zinc finger protein 493 |
| ARDS | Acute respiratory distress syndrome |
| NKG7 | Natural killer cell granule protein 7 |
| SPTA1 | Spectrin alpha, erythrocytic 1 |
| FGL2 | Fibrinogen like 2 |
| RGS2 | Regulator of G protein signaling 2 |
| IFI27 | Interferon alpha inducible protein 27 |
| VMP1 | Vacuole membrane protein 1 |
| SLPI | Secretory leukocyte peptidase inhibitor |
| PTX3 | Pentraxin 3 |
| TIMP1 | TIMP metallopeptidase inhibitor 1 |
| OLFM4 | Olfactomedin 4 |
| LCN2 | Lipocalin 2 |
| S100A9 | S100 calcium binding protein A9 |
| AKI | Acute kidney injury |
